# The androgen receptor-targeted proteolysis targeting chimera and other alternative therapeutic choices in overcoming the resistance to androgen deprivation treatment in prostate cancer

**DOI:** 10.1007/s12094-022-02957-x

**Published:** 2022-10-06

**Authors:** Liuxun Li, Jiangli Xu

**Affiliations:** 1 Solid Tumour Target Discovery Laboratory, Translational and Clinical Research Institute, Faculty of Medical Sciences, Newcastle University Centre for Cancer, Newcastle University, Newcastle upon Tyne, NE2 4HH UK; 2 Department of Pharmacy, No.921 Hospital of the Joint Logistics Support Force, Changsha, 410003 China

**Keywords:** Androgen receptor, Prostate cancer, Androgen deprivation treatment, Resistance, Proteolysis targeting chimera

## Abstract

Androgen receptor (AR) plays a vital role in prostate cancer (PCa), including castration-resistant PCa, by retaining AR signalling. Androgen deprivation treatment (ADT) has been the standard treatment in the past decades. A great number of AR antagonists initially had been found effective in tumour remission; however, most PCa relapsed that caused by pre-translational resistance such as AR mutations to turn antagonist into agonist, and AR variants to bypass the androgen binding. Recently, several alternative therapeutic choices have been proposed. Among them, proteolysis targeting chimera (PROTAC) acts different from traditional drugs that usually function as inhibitors or antagonists, and it degrades oncogenic protein and does not disrupt the transcription of an oncogene. This review first discussed some essential mechanisms of ADT resistance, and then introduced the application of AR-targeted PROTAC in PCa cells, as well as other AR-targeted therapeutic choices.

## Introduction

Globally, prostate cancer (PCa) is the second most frequently occurring cancer in men [1]. The human prostate is a single chestnut-like gland and located in the subperitoneal compartment beneath the bladder. Clinically, this gland is majorly divided into the transition zone, central zone, peripheral zone, and anterior fibro-muscular zone; most tumours emerge from the peripheral zone [2]. The orchiectomy was introduced to remove the testicles and subsequently retarded the progression of PCa in the 1940s [3]. The androgen receptor (AR) protein was later discovered, and it has been identified as a vital oncogenic protein activated by androgens, such as dihydrotestosterone (DHT), to stimulate the expression of AR-regulated genes such as prostate-specific antigen (PSA) and FK506-binding protein 5 (FKBP5) [4].

**Fig. 1:**
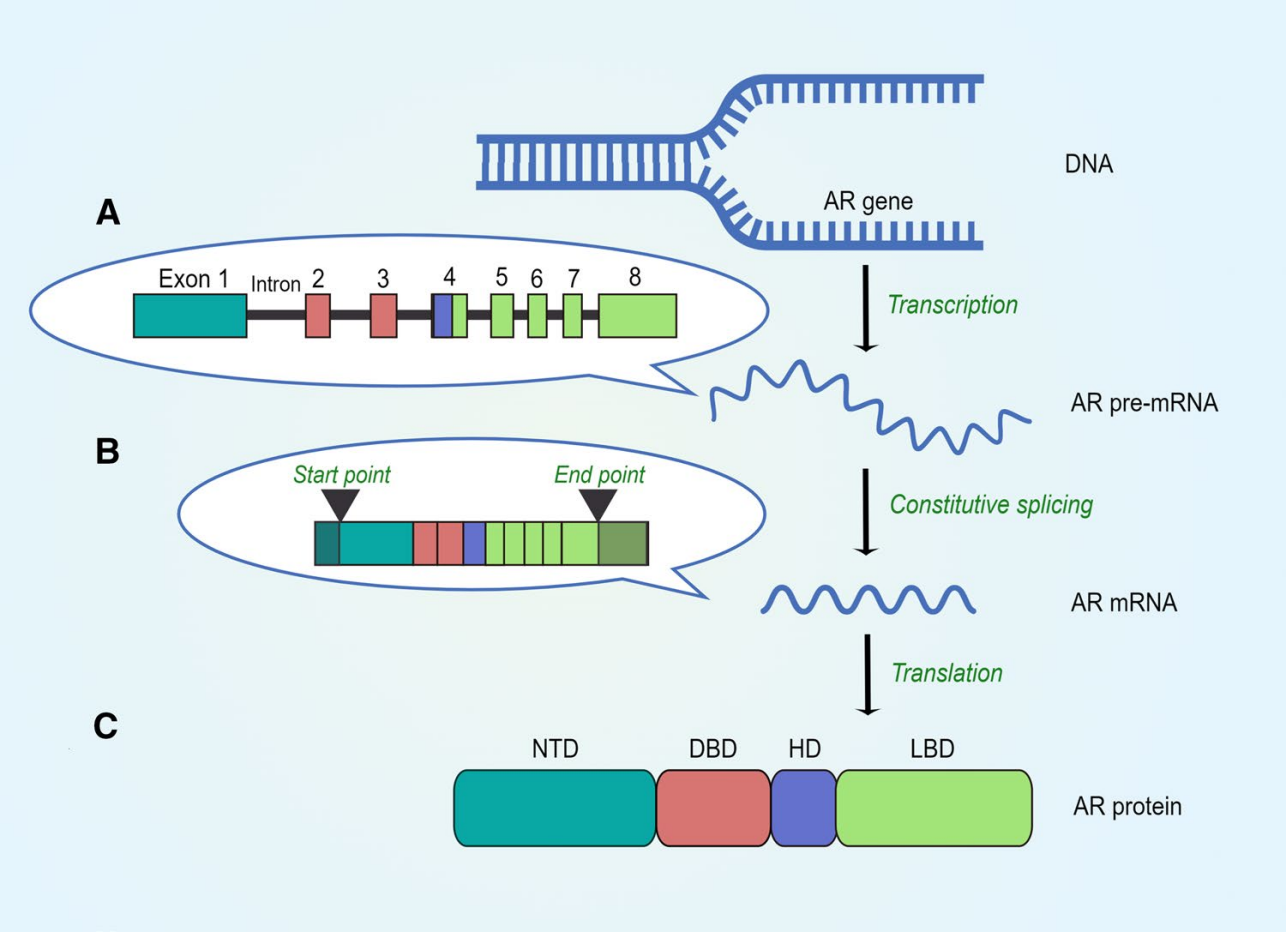
Schematic of AR protein synthesis. **A** The AR pre-mRNA, including eight exons and several introns, is generated from AR gene. **B** By constitutive splicing, all introns would be removed by multiple splicing factors, and mature AR mRNA containing eight exons is then produced. After mRNA being transferred into cytoplasm, the AR translation starts from exon 1 to exon 8. However, both exon 1 and exon 8 are not entirely utilised during the translation (the start point and end point were indicated in the graph). **C** The eight exons code for four distinct protein domains. Exon 1 codes for the NTD, exons 2 and 3 code for the DBD, exon 4 codes for the HD, and exons 4–8 code for the LBD (By Figdraw ID: AUWOY79b2a)

The AR belongs to the steroid receptor subfamily of nuclear receptors, which also includes glucocorticoid receptor (GR), progesterone receptor (PR), and estrogen receptor (ER) [5]. The AR gene lies on the X chromosome (q11–12) and is comprised of eight exons. Usually, these exons are interrupted by non-coding sequences, these sequences named introns which vary in size, and their lengths are usually much longer than exons [6]. Generally, the full-length AR protein is generated by constitutive splicing. In the beginning of AR protein synthesis, precursor message RNA (pre-mRNA), which contains introns and exons, would be produced from AR gene (Fig. 1A). Later, with the help of multiple splicing factors, the process of constitutive splicing turns pre-mRNA into mature AR mRNA by removing introns and joining the adjacent exons together (Fig. 1B). After that, the mature mRNA will be exported to cytoplasm and then translated into full-length AR protein which has four structural protein domains [7]. Among these four protein domains, the central DNA-binding domain (DBD) is encoded by exon 2 and exon 3, and it functions to bind specific DNA sequences found predominantly within regulatory regions of target genes. The DBD is a highly conserved region within steroid receptors. Adjacent to the DBD is a short flexible hinge domain (HD) which is encoded by exon 4. The HD functions as a bridge connecting the DBD and LBD and contains a nuclear localization signal which facilitates AR nuclear translocation. The C-terminal ligand-binding domain (LBD) is encoded by exons 4–8 and is less well conserved between steroid receptors and can bind androgens. The N-terminal domain (NTD) is encoded by the large exon 1. It is not only much longer than the equivalent domain of other steroid receptors, but also a relatively unstructured region [5, 8] (Fig. 1C).

Since AR and its signalling are of importance in PCa, androgen deprivation therapy (ADT) has been the key treatment for metastatic PCa for over 30 years [9]. The ADT aims to repress AR expression and abrogate AR signalling, and it currently includes luteinizing hormone releasing hormone (LHRH) agonists and AR antagonists (mainly targeting the LBD of AR) [10]. For example, in clinical practice, patients usually need to be injected with one LHRH agonist such as Triptorelin (11.25 mg), Goserelin (10.8 mg) or Leuprolide (22.5 mg) every 3 months, and to take one AR antagonist such as Apalutamide (240 mg), Darolutamide (600 mg), Bicalutamide (50 mg), Flutamide (750 mg), or Enzalutamide (160 mg) per day. ADT, via chemical or surgical castration, may result in remission in patients for 18–24 months; however, progression to castration-resistant PCa (CRPC) ultimately occurs after this period [11]. CRPC is now defined as a rising PSA concentration or disease progression despite androgen level in the castration range and accounts for most mortalities from PCa [12]. Interestingly, CRPC has been demonstrated to maintain a heavy reliance on AR signalling [13].

In this review, we first summarized some mechanisms of classical ADT failure which are AR pre-translational alterations and up-regulation of other nuclear receptors, and then extensively introduced one protein degradation technology named proteolysis targeting chimera (PROTAC) and its application targeting AR protein in PCa. Finally, some alternative AR-targeted therapeutic choices, including AR antagonist (targeting other domains of AR), androgen synthesis inhibitor, AR expression inhibitor, and AR agonist, were also discussed.

## Mechanisms of classical ADT failure

**Fig. 2:**
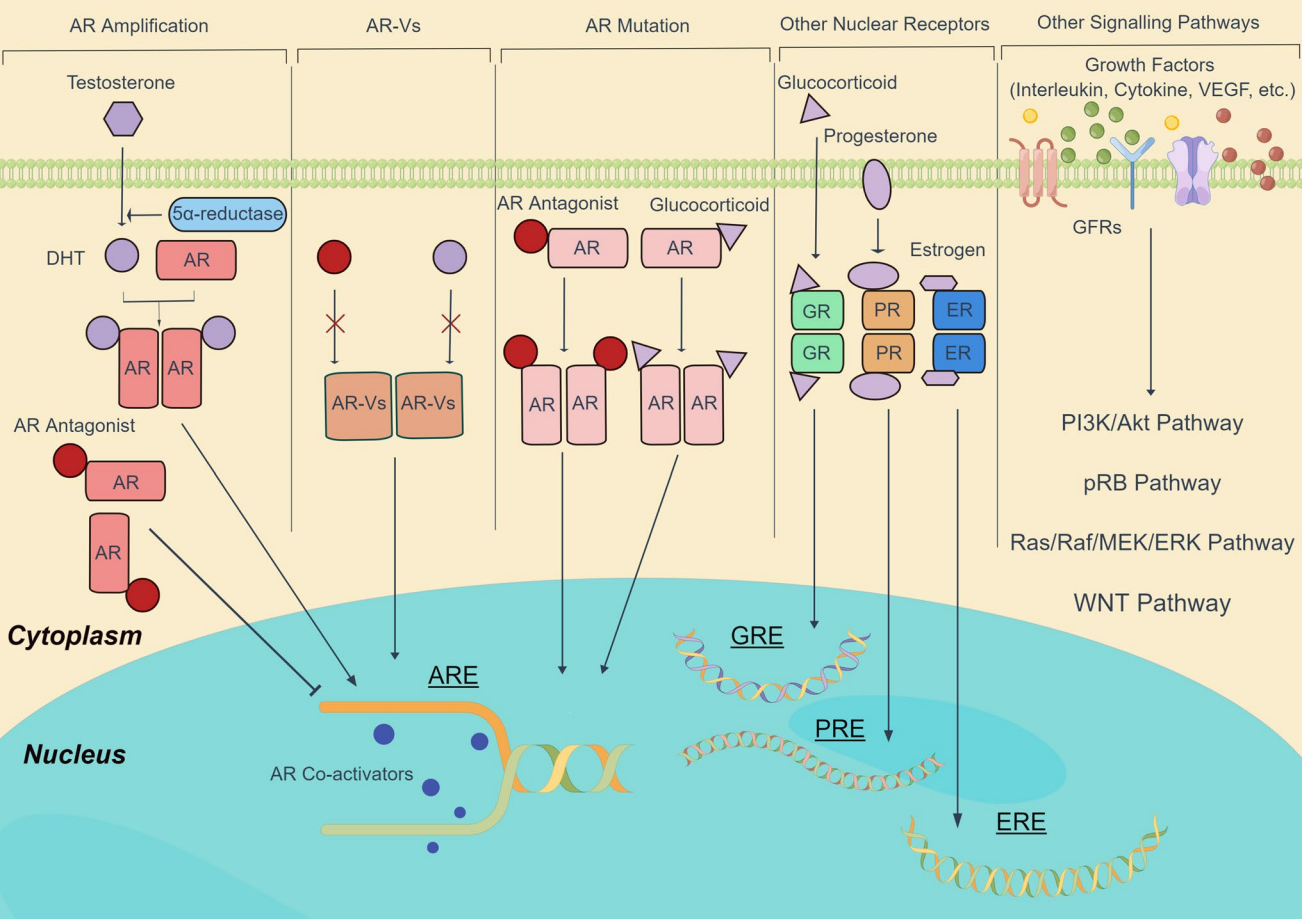
Schematic of mechanisms of classical ADT failure. These mechanisms could be divided into three categories which are AR-related mechanisms, up-regulation of other nuclear receptors, and other survival pathways. In details, AR amplification, AR mutation, and AR variants are three alternative processes to maintain AR signalling. Moreover, higher expression of GR, PR, or ER facilitates CRPC cell growth with distinct pathways. Finally, growth factors, such as interleukin, cytokine, and vascular endothelial growth factor (VEGF), are capable to bind growth factor receptors (GFRs) and activate various pathways, including PI3K/Akt, pRB, Ras/Raf/MEK/ERK, and WNT pathways. (By Figdraw ID: RSIRA4ca26)

In the past decades, mechanisms of classical ADT failure were gradually discovered (Fig. 2). AR amplification, AR mutation, and constitutively active AR variant (AR-V) are three alternative processes which help PCa cells continuously keep the AR signalling pathway activated. Moreover, other nuclear receptors or other survival pathways can replace the role of AR and continue to stimulate PCa cell growth.

### AR pre-translational alterations

#### AR amplification

By increasing the expression of AR protein, AR gene amplification promotes PCa progression and raises the chance of metastasis despite of reduced serum androgen level [14]. Two studies showed that upstream enhancers of AR gene and thereafter AR gene were amplified in androgen insensitive PCa patients. One recent study concluded the AR amplification existed in 81% of CRPC [15]. Another research indicated that 70–87% of CRPC patients contained amplification of AR, whereas the amplification was only detected in less than 2% of primary PCa [16]. More recently, Laura Porter et al. established two matched PCa patient-derived xenografts (PDXs). They first supported that AR amplification was observed in primary tumour by analysing PDX. Moreover, they noticed that the PDX with AR amplification was resistant to AR antagonists, such as Enzalutamide and Apalutamide. On the other hand, the PDX without AR gene amplification was sensitive to AR antagonists [17]. Several novel AR antagonists have been synthesized and recently tested in clinical trials, for example, Proxalutamide, TQB3720, and SHR3680 [18] (Table 1).

**Table 1:** The clinical trials of drugs which target AR pre-translational alterations in metastasized PCa patients in recent 5 years

Agents	Target	Additional drugs	Trial phase	Identifier	Start year
Targeting AR amplification					
Proxalutamide	AR	None	Phase 1	NCT02826772	2020
	AR	None	Phase 2	NCT05076851	2021
	AR	None	Phase 2	NCT03899467	2022
TQB3720	AR	None	Phase 1	NCT04853498	2021
SHR3680	AR	SHR3162	Phase 1	NCT02747342	2020
	AR	SHR3162	Phase 2	NCT04102124	2020
	AR	Docetaxel	Phase 2	NCT04603833	2021
Targeting AR mutation					
Enzalutamide	AR T877A	Flutamide	Phase 4	NCT02918968	2021
Apalutamide	AR T877A	ADT	Phase 3	NCT02489318	2022
Darolutamide	AR F876L	None	Phase 2	NCT01429064	2017
	AR W741L	Enzalutamide	Phase 2	NCT03314324	2021
	AR T877A	None	Phase 2	NCT02933801	2022
TRC253	AR F877L	None	Phase 1/2	NCT02987829	2021
Targeting AR-V					
Niclosamide	AR-V7	Enzalutamide	Phase 1	NCT03123978	2022

#### AR mutation

Similarly, AR mutations were rarely found in untreated PCa patients, and they were often found to emerge in patients who had been treated with anti-androgens for a long period of time [14]. The first identified AR mutation was the AR mutation (T877A) which is also the most frequently found mutation in PCa patients [19]. Later, more AR mutations had been discovered and analysed using gene sequencing [20]. Mutations in the LBD often cause a broadening of the ligand pocket; thus, AR protein can be activated by other hormones such as glucocorticoid and are also able to turn AR antagonist into mild agonist [21]. It is now well established that nearly 50% of AR mutations occurred in the LBD of AR, for example, AR H874Y, AR T877A, and AR L701H, approximately one-third of AR alterations, such as AR Q120E, AR A159T, and AR G216R, happened in the NTD [22]. Some second-generation AR antagonists, including Enzalutamide, Apalutamide, Darolutamide, and TRC253, were demonstrated to be capable to target some AR mutations (Table 1), however, they had less suppressive effects on other types of AR mutation and some agents even acted as AR agonist [21]. For example, the AR F877L mutation had been demonstrated to convert Enzalutamide into an agonist [23].

#### AR-V

In addition to AR amplification and AR mutation, the presence of AR-V was demonstrated to be a vital factor in ADT failure and AR-Vs were frequently found to be expressed in CRPC patients [8]. The first evidence of endogenous AR-V was found in CRPC cells, and the molecular weight varied between 70 and 80 kDa [24]. Currently, there are 30 AR-Vs identified that lack the LBD, including AR-V1, AR-V3, AR-V6, AR-V4, AR-V7, and AR-V9 [8]. These C-terminal-truncated variants are still able to drive AR signalling, as they are constitutively active in the absence of androgen [25].

Among discovered AR-Vs, AR-V7 is the most common variant found in clinical practice [26]. It is now established that AR-V7 mRNA contains exons 1–3 and cryptic exon 3 (CE3) [27]. The peptide sequence which originated from CE3 retained the ability for AR-V7 to localize into the nucleus [28]. Splicing factors, including Aurora A, SAP155, ASF/SF2, two DDX proteins named DDX39A and DDX39B, U2AF65 and long non-coding RNA PCGEM1, had been demonstrated to be involved in AR pre-mRNA alternative splicing and promoted AR-V7 mRNA formation [29–31]. Currently, one agent named Niclosamide was verified to inhibit AR-V7 transcription activity and its clinical value has been explored since early this year [32] (Table 1).

### Up-regulation of other nuclear receptors

#### GR

The GR, which is activated by glucocorticoids, is also known as nuclear receptor subfamily 3, group C, member 1 (NR3C1). Upon glucocorticoids binding, the GR proteins are translocated to the nucleus and interact with specific DNA regulatory sequences called glucocorticoid response element (GRE) to activate a specific subset of genes, including CDK1, SGK1, and FKBP5 [33]. It was reported that the level of GR protein decreased in PCa tissues compared with normal prostate tissues; however, higher expression of GR was observed in CRPC patients [34]. Moreover, rapid AR reduction following androgen deprivation resulted in GR up-regulation in several PCa cell lines [35]. One recent study suggested that the up-regulated GR expression was a consequence of an adaptive response after androgen inhibition. They noticed that abiraterone treatment was also capable to induce GR expression. This study further demonstrated that various subsets of PCa cells failed to form 3D-spheroids and were unable to growth as normal by reducing GR activity with Mifepristone which is a GR and PR antagonist [34]. However, one pre-clinical evidence illustrated that Mifepristone combined with Enzalutamide and Docetaxel had limited activity to inhibit bone metastasized PCa [36]. Moreover, one recent research concluded that the addition of Mifepristone to Enzalutamide failed to effectively reduce the level of PSA in CRPC patients in one recent clinical trial [37]. The GR antagonists being tested in clinical trials are listed in Table 2.

**Table 2:** The clinical trials of drugs which target three nuclear receptors in metastasized PCa patients

Agents	Target	Additional drugs	Trial phase	Identifier	Last update
Relacorilant	GR	Enzalutamide	Phase 1	NCT03674814	2018
CORT125281	GR	Enzalutamide	Phase 1/2	NCT03437941	2018
Mifepristone	GR and PR	None	Phase 2	NCT00140478	2009
		Enzalutamide	Phase 1/2	NCT02012296	2013
		Eribulin	Phase 1	NCT02014337	2018
Tamoxifen	ER	Casodex	Phase 2	NCT00637871	2011
		Bicalutamide	Phase 3	NCT00233610	2011
Toremifene	ER	None	Phase 3	NCT00129142	2013
		None	Phase 2	NCT00020735	2015
Raloxifene	ER	Bicalutamide	Phase 1	NCT01050842	2017
Fulvestrant	ER	None	Phase 2	NCT00476645	2014

#### PR

The PR, classified as nuclear receptor subfamily 3, group C, member 3 (NR3C3), is the most closely related steroid receptor to the AR. The PR displays 88% sequence homology to the AR in the LBD. There are two characteristic isoforms of the PR named PR-A and PR-B, both isoforms are stimulated by progesterone. Both receptor isoforms are encoded from a single gene. PR-B isoforms is a larger molecular weight protein having additional 164 amino acids at the N-terminal region compared with PR-A. The PR’s role in PCa tumourigenesis is currently well established. In PCa cells, Grindstad et al. found that high PR-B expression in PCa tumour tissue was associated with a worse prognosis, reflected in both biochemical and clinical failure [38]. Another study supported these findings and showed an up-regulation of the PR in progressing PCa patients [39]. To date, various studies strongly suggested that the up-regulated PR expression could be another potential mechanism contributing to the development of CRPC. It is possible that PR binds progesterone response element (PRE) and subsequently induces FKBP5, cyclin D1, and Ki67 expression, which are also AR-regulated genes, when AR activity was inhibited. In clinical research, the increased PR expression had also been demonstrated to induce and activate mutated forms of AR that emerged after long-term treatment with AR antagonists and the cytochrome P450, family 17, subfamily A, and polypeptide 1 (CYP17A1) inhibitor Abiraterone. In fact, increased progesterone was often seen in patients treated with abiraterone when CYP17A1 enzymatic activity was inhibited [40]. Although the effect of Enzalutamide combined with PR antagonist Mifepristone in recent clinical trial was limited [37], more specific GR antagonists combined with other AR antagonists or CYP17A1 inhibitor are needed to be explored in future clinical trials.

#### ER

ERα is alternatively classified as nuclear receptor subfamily 3, group A, member 1 (NR3A1). There are two distinct ER genes which are termed ERα and ERβ, the latter known as NR3A2. The role of ERα in advanced PCa was first described in 1999 by Bonkhoff and colleagues [41]. Since then, an increasing amount of evidence has been accumulated, demonstrating the impact of estrogen signalling pathways on prostatic carcinogenesis and PCa progression [42]. Later, estrogen was demonstrated in one study to be effective in treating metastatic PCa and is sometimes used as a secondary hormonal manipulation [42]. However, there are several studies, showing that estrogen stimulated the progression of PCa. For example, the expression of SGK1, which is characteristically regulated by AR, was also demonstrated to be increased upon estrogen response element (ERE) activation [43]. In clinical trials, agents, including Tamoxifen, Toremifene, Raloxifene, and Fulvestrant, had been tested in metastasized PCa (Table 2).

## Alternative AR-targeted therapeutic choices

### PROTAC

#### Introduction to PROTAC

In human cells, the ubiquitin proteasome system (UPS) plays a vital role in AR degradation and regulation. The UPS consists of ubiquitin (Ub), Ub-activating enzymes (E1), carrier Ub-conjugating enzymes (E2), Ub ligases (E3), deubiquitinating enzymes, and the 26S proteasome [44]. Biologically, researchers revealed that the hinge of the AR protein contains the conserved PEST (proline, glutamate, serine, and threonine motif) sequence which acts as a specific signal for protein degradation [45]. Aside from the involvement of the limited E1 and E2 enzymes, there are many E3 ligases, including mouse double minute 2 (MDM2), cereblon, carboxyl terminal of Hsp70-interacting protein (CHIP), ring finger protein 6 (RNF6), S-phase kinase-associated protein 2 (SKP2), speckle-type BTB-POZ protein (SPOP), tripartite motif protein 68 (TRIM68), and tumour susceptibility gene 101 (TSG101), that bind AR and had been demonstrated to be involved in AR protein degradation [46–52].

**Fig. 3:**
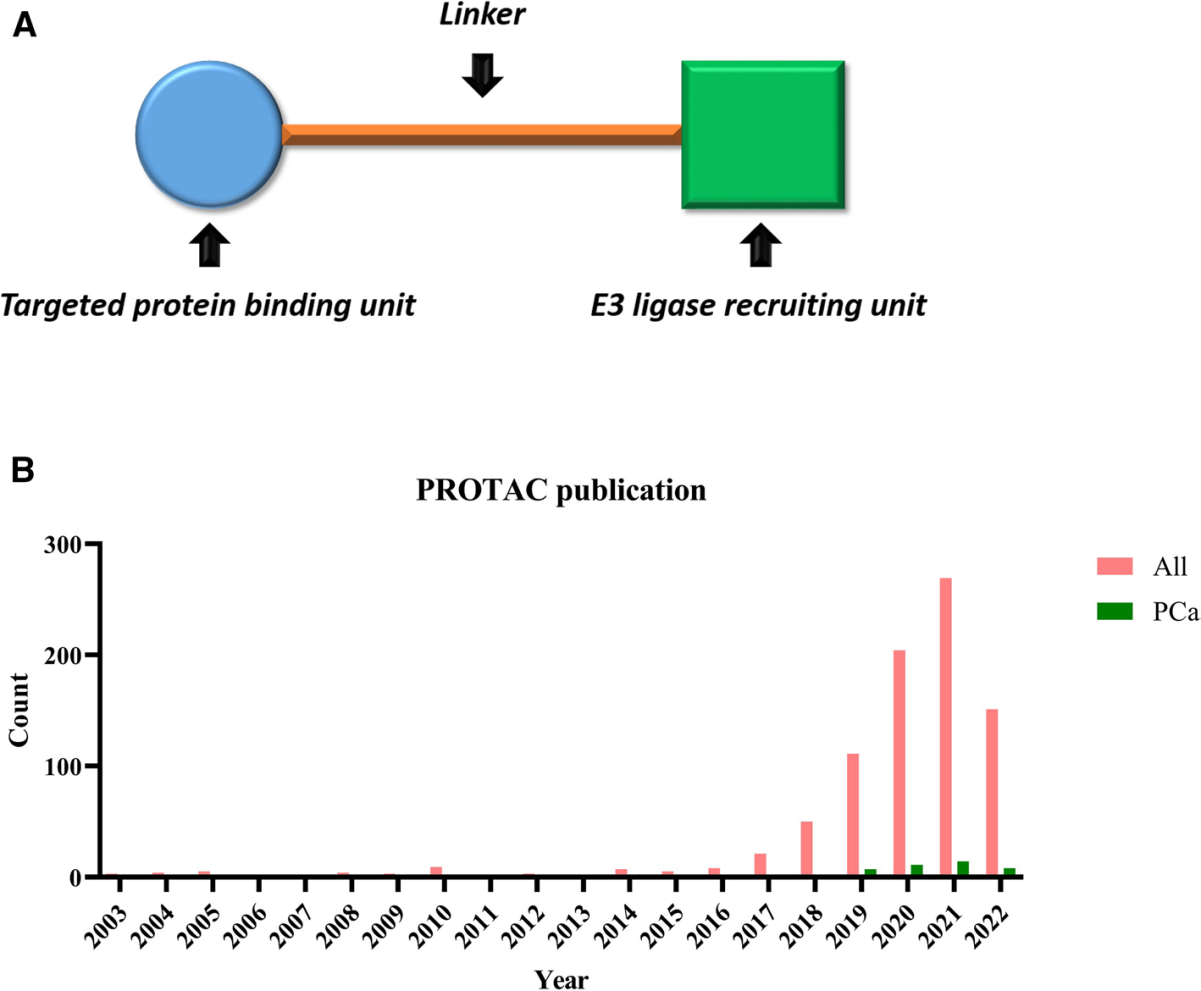
Introduction to PROTAC. **A** Illustration of PROTAC structure. A PROTAC comprises a specific binding unit to bind targeted protein, an E3 ligase recruiting unit, and one linker between them. **B** A graphical representation of the yearly growth in PROTAC publications from 2003 to 2022. Publications on all PROTACs and PROTACs in PCa were searched from PubMed (https://www.ncbi.nlm.nih.gov/pubmed). The literature was presented chronologically from 2003. Columns indicated the number of publications of all PROTACs and PROTACs in PCa

The PROTAC is a protein degradation technology that was extensively applied in recent years, it was designed to degrade its targeted oncogene protein through the UPS. Since AR protein mainly undergo systematic protein degradation via the UPS, the E3 ligases in this process can be harnessed by PROTAC. Structurally, PROTAC is comprised of a degradation machinery recruiting unit (usually an E3 ligase ligand), a targeted protein-binding unit and a linker region which joins these two components together [53] (Fig. 3A). The first PROTAC was introduced 20 years ago, this PROTAC comprised a methionine aminopeptidase-2 binding small molecule ovalicin attached through a linker (aminohexanoic acid) to an IκBα-based phospho-decapeptide recognition motif found within the E3 ligase SCF^β−TRCP^ [54]. Thereafter, the choice of design of PROTAC has gradually developed from a peptide-based PROTAC to a small molecule-based PROTAC. In the past 21 years, only a few articles focused on PROTAC before 2015; however, the number of PROTAC-related papers and reviews each year markedly increased after the year of 2018. Currently, the number of publications on PROTAC in PCa was relatively much lower than the whole publications (Fig. 3B).

Recently, another novel protein degradation technology called autophagy-targeting Chimera (AUTOTAC) is proposed to eliminate a protein of interest through the autophagy–lysosome system. The AUTOTAC is designed to bind a targeted protein and p62 which is an activator of autophagy. In early this year, one AUTOTAC named VinclozolinM2-2204 composed of a p62-binding moieties and vinclozolinM2, was synthesized to degrade AR protein. The vinclozolinM2 is a metabolite of vinclozolin and it prevents androgen from binding the LBD of AR protein. This AUTOTAC had been demonstrated to decrease the level of AR protein successfully at 0.5–10 µM concentrations in LNCaP cells [55]. Unlike the UPS which degrades small unfolded and misfolded proteins, the autophagy–lysosome system digests a broader range of large substrates, including insoluble mutant protein, degenerated mitochondria, and peroxisomes [56]. Although the idea of AUTOTAC was newly proposed and its application is currently limited, it is still promising for AUTOTAC to synergize with PROTAC in the depletion of AR protein in the near future.

#### Mechanism of PROTAC

The molecular mechanism of PROTAC is different from traditional drugs that usually function as inhibitors or antagonists to disrupt the transcription of an oncogene or competitively bind specific oncogenic protein, resulting in the suppression of tumour cell proliferation and retarding cancer progression. PROTAC, on the other hand, is specifically designed to join its targeted protein and one selected E3 ligase together. More specifically, the PROTAC binds its targeted protein and recruits an E3 ligase via ligand motif after entering the cells (Table 3).

**Table 3:** Current AR-targeted PROTACs reported in PCa research

Agents	Target ligand	E3 ligase	References
PROTAC-2	DHT	SCF^βTRCP^	[63]
PROTAC-5	DHT	VHL	[64]
Protac A	SARM	MDM2	[65]
DHT-PROTAC	DHT	VHL	[66]
Compound 13	DHT	cIAP	[67]
Compound 42a	AR antagonist	cIAP	[68]
ARCC-4	Enzalutamide	VHL	[69]
ARD-69	AR antagonist	VHL	[70]
ARD-266	AR antagonist	VHL	[71]
ARD-61	AR antagonist	VHL	[72]
PAP508	RU-59063	Thalidomide	[73]
ARV-110	AR agonist	Cereblon	[74]

Some target ligands of these PROTACs are not mentioned in the publication

**Fig. 4:**
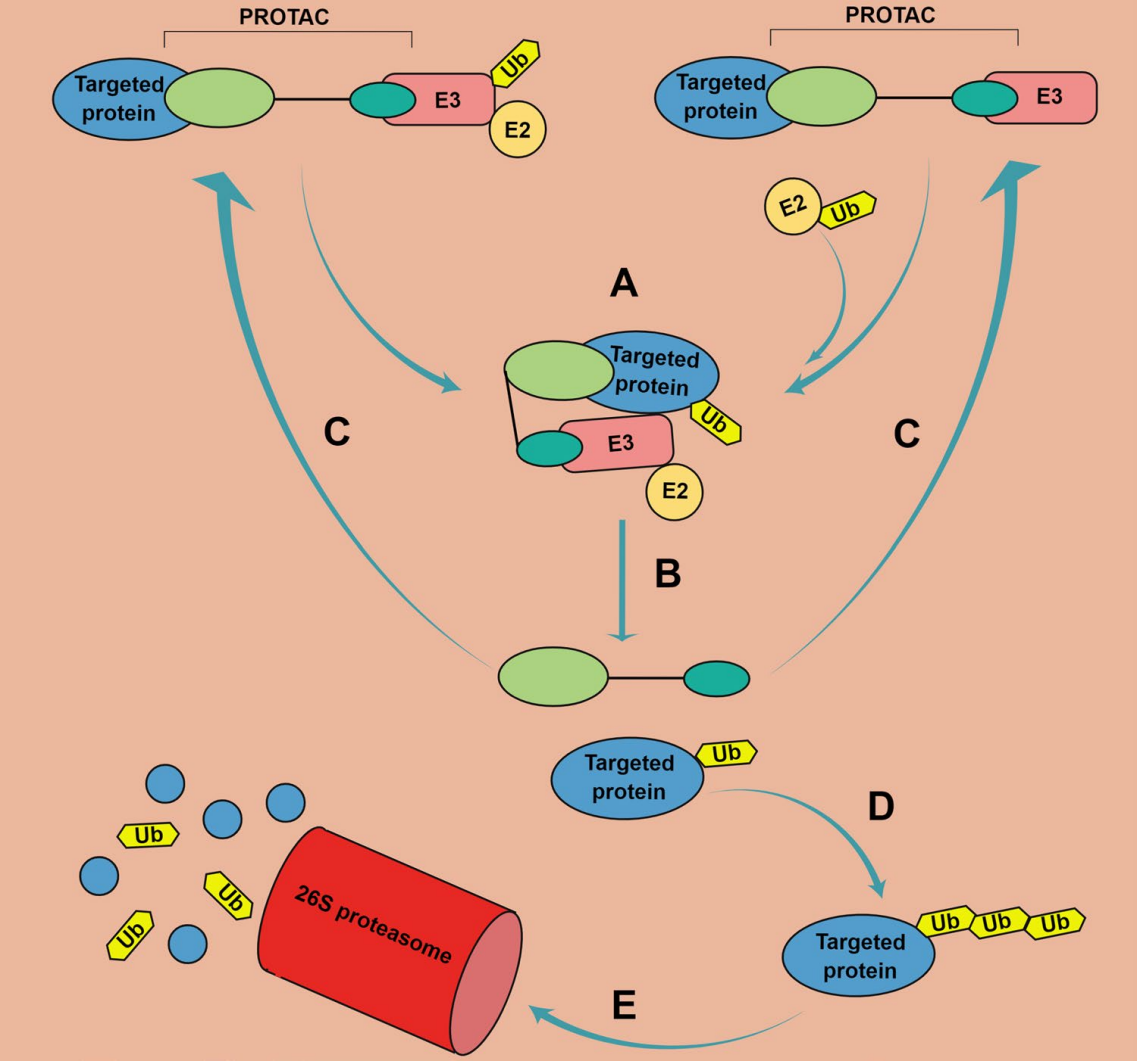
Schematic of mechanism of PROTAC action. **A** PROTAC first functions like a bridge between its targeted protein and E3 ligase (being either untagged or tagged combined with E2) through binding both with its two ends. Then, the Ub is transferred to the substrate protein after E3-binding protein substrate. **B** The PROTAC and Ub-tagged substrate are subsequently released from the complex. **C** The unmodified PROTAC can be restored and repeatedly performs its function. **D**, **E** The mono-Ub-tagged substrate can add additional Ub linked with isopeptides until the poly-Ub-tagged substrate is recognized by the 26S proteasome, resulting in suppression of oncogene expression. (By Figdraw ID: OISIAefe92)

Since untagged E3 ligase is recruited to interact with its potential substrate, E3 ligases in the Cullin-RING family, such as MDM2 and cereblon, are more qualified and suitable for E3 ligase recruitment unit design. Therefore, a PROTAC acts as a bridge to join its target and an E3 ligase together. Subsequently, the Ub is transferred onto the targeted substrate protein and this Ub-tagged substrate will be dissociated from the E3 ligase. PROTAC, at this stage, is also released due to the structural changes of both proteins. The single Ub-tagged substrate requires another ubiquitinated E3–E2 complex to add additional Ub molecules. This process usually repeats several times until the poly-Ub-tagged substrate protein can be recognized by the 26S proteasome [57]. Finally, the ‘temporarily free’ PROTAC can be restored and is able to conjugate to another targeted protein molecule and repeat the process of protein degradation, multiple cycles of recruitment, and degradation can occur (Fig. 4).

#### AR-targeted PROTACs

Small molecule PROTACs have great potential as anti-PCa drugs with several advantages over anti-androgen therapies. For example, PROTAC can operate to destroy rather than inhibit the activity of the AR protein that may minimize the inductions of pre-translational alterations such as AR mutations and AR-Vs. Additionally, lower concentrations of PROTAC are suitable because of its continuous rounds of AR protein degradation [58]. In the past 2 decades, increasing number of PROTACs had been synthesized in the area of PCa. In addition to those PROTACs based on targeting BRD4, CDK, and others, the AR protein remains to be a key target of PROTAC action [59, 60] (Table 4).

**Table 4:** Clinical trials on different AR-targeted therapies in recent 5 years

Agents	Additional treatment	Trial phase	Identifier	First post
PROTAC				
ARV-110	Enzalutamide, Abiraterone	Phase 1/2	NCT03888612	2019
	Abiraterone	Phase 1	NCT05177042	2022
AR antagonist (targeting other domains of the AR)				
EPI-506 (NTD)	None	Phase 1/2	NCT02606123	2015
Androgen synthesis inhibitor				
Abiraterone	ADT, Prednisone	Phase 3	NCT01715285	2012
	Olaparib	Phase 2	NCT01972217	2013
	ADT, Docetaxel	Phase 3	NCT01957436	2013
	Apalutamide	Phase 3	NCT02257736	2014
	Ipatasertib, Prednisolone	Phase 3	NCT03072238	2017
Orteronel	Prednisone	Phase 3	NCT01193244	2010
	None	Phase 3	NCT01707966	2012
Galeterone	None	Phase 2	NCT01709734	2012
	None	Phase 3	NCT02438007	2015
AR agonist				
Testosterone	Nivolumab	Phase 2	NCT03554317	2018
	Carboplatin	Phase 2	NCT03522064	2018
	Darolutamide	Phase 2	NCT04558866	2020
	Rucaparib	Phase 3	NCT04455750	2020
	Enzalutamide	Phase 2	NCT05081193	2021
	None	Phase 2	NCT05011383	2021
	Olaparib	Phase 2	NCT03516812	2022
Fluorinated DHT	None	Phase 1	NCT01724619	2012

In 2003, Sakamoto and his colleagues first introduced an AR-targeted peptide PROTAC. This DHT-based PROTAC was introduced into HEK293^AR−GFP^ cells. Green fluorescence protein-AR was observed to disappear within 1 h, illustrating that this PROTAC degraded the AR protein [61]. Since then, some new AR-targeted PROTACs which vary in the target ligand, the recruited E3 ligase and structure/length linkers have been synthesized and tested. Among all AR-targeted PROTACs, the often-chosen targeted ligands were DHT and AR antagonist, and the recruited E3 ligase were Von-Hippel–Lindau (VHL) and cellular inhibitor of apoptosis (cIAP) (Table 1).

Previously, investigation on protein degradation stimulated by different concentrations of PROTAC had identified a phenomenon named hook effect. This effect essentially demonstrates that the degradation of protein would be diminished at high concentrations of PROTAC [62]. Among publications on AR-targeted PROTAC, one study indicated the hook effect in PCa cells treated with a PROTAC named PAP508. This AR-targeted PROTAC is based on RU-59063 which is an AR activator, it was chosen for its high selectivity and ability to target AR protein. They noticed that the optimal concentration for AR degradation in VCaP cells, which express full-length AR and AR-V7 proteins, was approximately 10 μM; however, less degradation was observed at the lower concentration, 5 μM and the higher dose, 20 μM of PROTAC. They also discovered that the optimal concentration of PAP508 is different between VCaP and LNCaP cells which is androgen responsive with mutated (T877A) AR but no AR-Vs [63].

As testosterone at low level still exists in men under castration condition, the ability of PROTAC to compete with androgen to bind the LBD of AR is of importance [64]. Among those reported AR-targeted PROTACs, 1 μM of ARCC-4 was found to deplete AR proteins in VCaP cells for 6 h; however, ARCC4 combined with 1–10 nM of synthetic androgen R1881 failed to degrade AR protein [65]. Another study illustrated that 100 μM of DHT-PROTAC described by Yue-Qing was capable to compete with 10 nM DHT; however, its AR degradative property was lost when 100 μM or higher concentrations of DHT was included [66].

Regarding the initial time point at which AR degradation occurs mediated by PROTAC, Da et al. also explored AR degradation in a time-dependent manner. The PROTAC, PAP508 began to reduce AR protein after 12 h and its maximum effect occurred at around 16 h [63]. Although no time-dependent experiments were performed, another study showed that a DHT-based PROTAC degraded AR protein at 4 h [66]. Additionally, 100 nM of ARD-69 (produced by Han et al.) significantly reduced the level of AR protein at 2 h in both LNCaP and VCaP cells, and then reached near-complete AR depletion after 4 h treatment [67]. Similarly, 10 nM of ARV-110 also degraded AR protein at 2 h in the VCaP cells [68]. In another study by Han, the AR degradation mediated by 100 nM of ARD-266 or ARD-61 occurred at 1 h and AR depletion happened after 3 h treatment. The structural difference between ARD-266 and ARD-61 relates to the affinity for the VHL E3 ligase. They suggested that even PROTAC with low-affinity ligand for VHL is still capable of degrading AR protein and its effect is similar to a PROTAC with a high-affinity ligand [69].

### Androgen synthesis inhibitor

To suppress AR signalling, ADT has been globally used in treating metastasized PCa patients. Although effective in eliminating the majority of androgen produced in the testes, androgen can still be synthesized intratumorally and by the adrenal gland [70]. Abiraterone is the first androgen synthesis inhibitor to target adrenal androgen production [71]. It functions to block the enzyme CYP17A1 which exists in testis and adrenals and therefore results in substantially reducing androgens [72]. More recently, further highly selective androgen synthesis inhibitors, such as Orteronel and Galeterone, were synthesized and advanced to clinical trials [73, 74].

### AR expression inhibitor

Different from the mechanism of PROTAC to target AR protein, another type of therapy termed AR expression inhibitor utilised antisense oligonucleotides to target AR mRNA by binding the complementary region of AR mRNA which results in significant reduction of mRNA transcription [75]. One example of an AR expression inhibitor is ENZ-4176, which was tested in its clinical phase 1 study in CRPC patients. However, this clinical trial conducted by two institutions (Royal Marsden NHS Foundation Trust and The Institute of Cancer Research and Memorial Sloan-Kettering Cancer Centre) not only recruited limited patients, but it suggested several toxicities including fatigue and reduced liver function after taking ENZ-4176, which eventually resulted in suspending the trial [76].

### AR antagonist (targeting other domains of the AR)

Since current AR antagonists which target the LBD of AR often induced AR mutations and AR-Vs, several AR antagonists were designed to target the NTD or DBD of AR protein. EPI-001 and EPI-506 are two representative NTD inhibitors. Both EPIs were able to greatly suppress the growth of PCa cells which expressed AR, AR mutations, or AR-Vs via inhibiting the transcriptional activity [77–80]. Aside from the NTD, the DBD had also been potential target for its vital role in protein dimerization. For example, VPC-17005 and VPC-14449 are two DBD inhibitors which aimed at disrupting AR and/or AR-Vs dimerization and chromatin localization [81, 82].

### AR agonist

The level of androgen has long been regarded as an important factor for PCa progression [83]. The level or concentration of bioavailable testosterone in older men is lower than that of younger men; however, the incidence of PCa in older men is significantly higher than that in younger men [84]. Back in the 1940s, the necessity to eliminate androgens in PCa patients was first introduced by Huggins; thereafter, this treatment method was verified and supported by subsequent studies [85]. Nonetheless, as clinical complications arose and ADT failures continued, the role of androgens in treating PCa cells has been revaluated by many studies in recent years [86]. First, two reports suggested that testosterone therapy is safe for untreated PCa patients who received active surveillance [87, 88]. Moreover, it had been illustrated that testosterone replacement was capable to reduce the risks of PCa recurrence. One of these studies showed that although additional testosterone induced higher levels of PSA in patients who had undertaken radical prostatectomy or radiation therapy, it reduced the recurrence of PCa compared with the control group [89]. Another clinical prospective study, however, noticed no significant increase in PSA expression in post-radical prostatectomy patients who treated with testosterone [90]. Correspondingly, a few studies showed that testosterone may play a protective role in high-grade PCa patients; however, more large-scale clinical trials are required [91]. Furthermore, one recent paper investigated the changes in AR and AR-V7 mRNAs’ expression and DNA repair capability in CRPC xenografts derived from Enzalutamide-resistant patients after treating them with high-dose testosterone. They concluded that testosterone treatment robustly suppressed both AR and AR-V7 mRNAs’ expression and DNA repair activity [92]. As treatment option beyond Enzalutamide is limited, high-dose testosterone therapy presents another potential choice for further treatment.

## Conclusions and future perspective

In summary, ADT currently remains the first choice for advanced PCa patients; however, ADT failure often happens and presents with resistance, defined as CRPC [12]. The resistance to ADT remains a tremendous issue in CRPC treatment. In the past decades, researchers explored some therapeutic strategies aiming at overcoming or bypassing ADT resistance. Among these strategies, PROTAC, AR antagonist (targeting other domains of the AR), and AR agonist could be promising therapeutic choices in the future.

PROTAC has been massively investigated during the last 5 years. Inspiringly, ARV-110 has been used in two clinical trials (NCT03888612 and NCT05177042) which started from 2019 and 2022. Although PROTAC selectively degrades AR proteins regardless of pre-translational alterations, there are still several challenges such as off-target, potentially resistance to protein degradation, and structural and functional stability. To overcome these challenges, some potential studies need to be done in the future. In one recent study, ARCC-4 was demonstrated to slightly reduce the level of AR-V7 protein in VCaP cells which suggests that AR-targeted PROTAC may have potential ability to degrade AR-V7 as well [68]. Apart from AR-Vs, other potential targets of AR-targeted PROTAC, including AR co-activators, AR co-repressors, and other nuclear receptors, could also be further investigated. To explore the unknown resistance to PROTAC, some PCa cell lines can be treated with dimethyl sulfoxide, PROTAC or one AR antagonist such as enzalutamide, and passed down for 1 month or more. Moreover, since barely recent synthesized PROTAC had been used to treat PCa cells or xenografts for long-term treatment, the functional and structural stability of PROTAC may need to be evaluated in a long period of time. For AR antagonist (targeting other domains of the AR), it is necessary to explore whether such AR antagonist would induce consequential resistance in CRPC cells. Moreover, there are several aspects to be carefully considered after CRPC patients intaking AR agonists, such as testosterone. For example, how high dose of AR agonist influences the physical condition of CRPC patients, what dose range of AR agonist can be safe and tolerant to the patients.

In short, the effects of three alternative therapeutic choices need to be extensively evaluated in both treatment-naïve PCa and AR-positive CRPC patients in clinical trials and following cases. Other alternative anti-AR therapies, however, may play less-satisfied roles in CRPC. For example, androgen synthesis inhibitor, such as abiraterone, had already been shown to induce resistance [93]. On the other hand, ENZ-4176, one AR expression inhibitor, had been demonstrated to cause severe toxicities [76].

## Data Availability

It is not applicable.
